# Age at natural menopause and development of chronic diseases in the female population of Kharameh, Iran: A historical cohort study

**DOI:** 10.1002/hsr2.2042

**Published:** 2024-04-21

**Authors:** Zahra Pasokh, Mozhgan Seif, Haleh Ghaem, Abbas Rezaianzadeh, Masoumeh Ghoddusi Johari

**Affiliations:** ^1^ Student Research Committee, Department of Epidemiology, School of Health Shiraz University of Medical Sciences Shiraz Iran; ^2^ Non‐Communicable Diseases Research Center, Department of Epidemiology, School of Health Shiraz University of Medical Sciences Shiraz Iran; ^3^ Colorectal Research Center, Department of Epidemiology, School of Health Shiraz University of Medical Sciences Shiraz Iran; ^4^ Breast Diseases Research Center, Community Medicine Department Shiraz University of Medical Sciences Shiraz Iran

**Keywords:** age, chronic diseases, menopause, post‐menopausal period

## Abstract

**Background and Aims:**

Declines in estradiol levels after menopause have been reported to be associated with several health outcomes. This study aimed to determine the effect of age at natural menopause (ANM) on some of the most common chronic diseases.

**Methods:**

This historical cohort study was performed on 2636 postmenopausal women aged 40–70 years participating in phase one of the PERSIAN cohort study in Kharameh, Iran, during 2015–2017. The effect of early (<45 years), intermediate (45–53 years), and late menopause (>53 years) on chronic diseases such as hypertension, diabetes, ischemic heart diseases, stroke, thyroid diseases, and depression was assessed using classic logistic regression for diseases with an incidence rate of more than 10% and Firth's logistic regression for diseases with an incidence of less than this amount.

**Results:**

The mean age of women was 53.48 ± 8.59. Respectively, early and intermediate menopause was associated with ischemic heart disease (odds ratio [OR = 1.61, 95% confidence interval [CI]: 1.08–2.42; *p* = 0.020), (OR = 1.57, 95% CI: 1.13–2.21; *p* = 0.008) and thyroid diseases (OR = 3.10, 95% CI: 1.64–6.24; *p* < 0.001), (OR = 1.83, 95% CI: 1.02–3.57; *p* = 0.042). furthermore, early menopause was a risk factor for diabetes (OR = 1.46, 95% CI: 1.07–2.00; *p* = 0.018), depression (OR = 4.79, 95% CI: 2.20–11.79; *p* = <0.001) and stroke (OR = 3.00, 95% CI: 1.08–9.32; *p* = 0.034).

**Conclusions:**

In this study, women with diabetes, ischemic heart diseases, stroke, thyroid disorders, and depression had a younger ANM compared to their healthy counterparts. Therefore, applying appropriate strategies to postpone the age of menopause, can reduce the incidence of these types of chronic diseases.

## 1 INTRODUCTION

It is reported that menopause is associated with numerous health problems such as chronic diseases.^1^, ^2^ Natural menopause is defined as the permanent cessation of menstruation for 12 months without any pathological or physiological cause.^3^ Menopause, which is associated with the cessation of the secretion of estrogen and progesterone hormones by the ovaries, is an important event in women's lives.^4^ Some effects of decreasing ovarian estrogen production during menopause are as follows: changes in connective tissues, cardiovascular diseases, metabolic disorders, obesity, urogenital ailments, sleep disorders, and psychiatric disorders such as sudden mood changes, depression, problems in coping with stressful situations, fatigue, nervousness, and memory loss.^5^

The age of menopause varies in different countries and ethnicities due to differences in reproductive history, lifestyle, socioeconomic conditions, and genetic and environmental factors.^6^ The median age of the world's population is increasing due to the decline in fertility and the increase in life expectancy.^7^ Since the average age of menopause is 51 years, more than one‐third of a woman's life is spent after menopause.^8^ It has been observed that the age of menopause is lower in developing countries than in developed countries.^9^ The age of menopause in Iran is reported to be 48.57 years.^10^ The onset of menopause before the age of 45 is stated as early menopause,^11^ however, late menopause differs between 52 and 55 years of age in different investigations.^2^, ^12^, ^13^

Cardiovascular diseases, mainly ischemic heart disease and stroke are the leading cause of global mortality and one of the main causes of disability.^14^ The postmenopausal period has been reported as a risk factor for cardiovascular diseases.^15^ But the results of previous studies are still inconsistent. In a longitudinal study of 568 women, menopause did not affect hypertension and cardiovascular disease; and they were solely attributed to their older age.^16^ However, in a cohort study of 144,260 postmenopausal women in England, early menopause was associated with an increased risk of cardiovascular disease (including stroke and coronary heart disease).^17^ The effect of menopause on the incidence of diabetes and hypertension, which are risk factors for cardiovascular diseases, remains controversial. In a cross‐sectional study conducted in Japan, early menopause was associated with coronary heart disease, but there was no relationship between the age of menopause and the prevalence of stroke, hypertension, and diabetes.^18^ A meta‐analysis study found that early menopause increases the risk of developing diabetes compared to women who go through menopause after age 45.^19^ In the study of Izumi et al.,^15^ younger age at menopause and longer postmenopausal period were associated with increased blood pressure.

Depression is the greatest cause of global disability and the main cause of suicide mortality.^20^ Women are more vulnerable to depressive disorders than men.^21^ Meanwhile, depression is one of the most important problems among menopausal women.^22^, ^23^ In a meta‐analysis study conducted by Georgakis et al., it was observed that longer exposure to endogenous estrogens, such as older age at menopause and a longer reproductive period, is associated with a lower risk of depression in later life.^24^ Nonetheless, in some studies, there is no relationship between menopausal age and depressive symptoms.^25^

The incidence of thyroid diseases in women is 5–20 times higher than in men. In addition, the prevalence of thyroid diseases increases with age. Accordingly, hypothyroidism, nodular goiter, thyroid autoimmunity, and cancer often occur in postmenopausal and elderly women.^26^ Although there is limited research on this issue, some studies found a lower age of menopause in patients with thyroid diseases.^27^, ^28^

Given that the age of menopause in Iran is below the global average and hormonal fluctuations can impact disease development, in this study, the risk of some diseases after the occurrence of menopause was investigated.

In addition, in Iran, few studies have been conducted in this field; and in most of them, chronic diseases are assumed as determinants of menopause age.^29^ In the current study, we have investigated whether age at natural menopause (ANM) can be a risk factor for some common chronic diseases such as hypertension, diabetes, ischemic heart diseases, stroke, thyroid diseases, and depression among women aged 40–70 years.

## 2 METHODS

### 2.1 Design and participants

This historical cohort was conducted on 2636 postmenopausal women aged 40–70 years who participated in the first phase of the PERSIAN cohort study in Kharameh City, located in the Fars province of Iran from 2015 to 2017. The PERSIAN (Prospective Epidemiological Research Studies in Iran) is a national cohort study started in 2014 by the Ministry of Health and Medical Education to encourage research in the fields of medicine, epidemiology, health, and nutrition. The Persian cohort includes different geographical, climatic, and ethnic groups in 18 provinces of the country.^30^ Data collection in the first phase of the PERSIAN Kharameh cohort study started in 2015 and ended in 2017.

Previously, the whole residents of Kharameh aged 40–70 years with Iranian nationality were enrolled in the PERSIAN cohort study; but the entry of deaf, blind, dumb, and mentally retarded people who were unable to refer to the cohort center was refused. Eligible people were included in the Kharameh cohort study through the census, and after obtaining their informed consent, standardized questionnaires containing demographic information, history of diseases, menopause status and ANM were completed through a face‐to‐face interview, and anthropometric information such as height and weight were measured in person by trained staff. It is worth mentioning that the validity and reliability of the questionnaires have been checked in advance and mentioned in the PERSIAN cohort study.^30^ Among 5944 women participated in the Khrameh cohort study, 2636 women who experienced menopause naturally included in the current study. Since we aimed to determine the effect of the ANM on six chronic diseases, in each dataset, we used the information of women who reported to have hypertension, diabetes, ischemic heart diseases, stroke, thyroid disorders, and depression after the occurrence of menopause.

### 2.2 Variables

To assess the effect of ANM on the incidence of the mentioned diseases, the ANM of patients was compared with their healthy counterparts. In analyses, each disease was considered as the dependent variable and ANM was considered as the independent variable. The ANM was divided into three levels: early menopause (less than 45 years old), moderate (45–53 years old), and late (more than 53 years old) based on the 20th and 80th percentiles, which is consistent with Lee et al.'s^31^ study.

Body mass index (BMI), smoking (yes or no), and demographic variables such as age, education years, residence (city or village), job (employee or housewife), and socioeconomic status (low, moderate, high, and very high) were included in the analysis as confounding factors. It is noteworthy that socioeconomic status is calculated by Principal Component Analysis using information such as homeownership, home size, number of bathrooms in the house, owing cars and their prices, domestic and international travels, reading books, access to the internet, owning mobile phones, computers, televisions, washing machines, dishwashers, refrigerators, vacuum cleaners, and microwaves.

#### 2.2.1 Assessment of menopausal status and ANM

To assess the menopausal status of women and their ANM, women were asked: (1) Have you reached menopause? (Options: Yes or No) (2) At what age did you experience menopause? (3) Have you naturally gone through menopause? (Options: Yes or No).

In addition, we assessed their responses to questions regarding the history of hysterectomy and oophorectomy and their age at these procedures, as well as their current pregnancy status, to ensure the accuracy of responses related to natural menopause. Actually, the natural menopause variable has also been checked and reviewed according to the answers to these questions. Among 5944 women, 3188 people reported to have reached menopause; among these menopausal women, we excluded 97 women who reported having unnatural menopause, 35 women due to current pregnancy, 412 women due to hysterectomy and 8 women due to oophorectomy before the occurrence of menopause. So, 2636 women were experienced menopause naturally. We had no missing in variables of menopausal status, menopausal age, and history of chronic diseases; Although the smoking status of an individual was missing.

### 2.3 Ethical considerations

This study was approved by the Ethics Committee of Shiraz University of Medical Sciences (IR.SUMS.SCHEANUT.REC.1400.104). Informed consent was obtained from all participants before participation. The participants were assured of confidentiality and anonymity.

### 2.4 Statistical methods

The analysis was done by R software version 4.2.0 with a two‐tailed significance level of 0.05. We applied classic logistic regression for diseases with an incidence rate of more than 10% and Firth's logistic regression (using the “logistf” package) for diseases with an incidence of less than this amount. It is worth mentioning that for binary responses, the rarity of the event leads to a large variance of the estimators, accordingly, the power decreases. Therefore, to prevent this problem in estimations, when the event was rare, Firth's logistic regression was utilized for modeling. Variables were selected by stepwise method and based on Akaike Information Criterion (using the “MASS” package). For a better understanding of classic and Firth's logistic regression, we briefly review these approaches. In addition, in crude analysis, we used Chi‐squared and analysis of variance test.

#### 2.4.1 Classic logistic regression

One of the most widely used types of regression in medical sciences is logistic regression.^32^, ^33^ Logistic regression is often used to assess the relationship between a binary outcome and a set of independent variables. This relationship can be interpreted using an odds ratio (OR) with a 95% confidence interval (CI).

If Y_i_ = (i = 1, …, n) is a binary outcome for ith participant that obeys a Bernoulli distribution with the binomial probability π_i_ = Pr(Yi =1), the model of logistic regression will be $\log\left(\frac{\Pi_{i}}{1-p_{i}}\right)=\beta^{T}x_{i}$. Where β_T_ = (β_0_, β_1_, …, β_k_) is the regression coefficients and xi= (1, x_i1_, …, x_ik_)^T^ is the predictor values for the i'th participant. Usually, β is estimated by the maximum likelihood (ML) method. Generally, the CIs of β are obtained via Wald's method or profile likelihood. In sparse datasets, profile likelihood‐type CI outperforms Wald‐type CI due to the failure of the normal approximation incorporated in its calculation.^34^ Therefore, we use the CI of the profile likelihood. 100 (1 − α) %CI is all values β_j_ (j = 0,…, k) where at the significance level of α, the generalized likelihood ratio test of the null hypothesis H_0_: β_j_ = β_j0_ is not rejected.^35^ The ML estimation of coefficients can be significantly biased upward or downward when there are a small number of study participants on the levels of outcome and independent variable or when there is an unbalanced structure of dependent or independent variables.^36^ This bias is noticed as small sample bias or sparse data bias.^37^ This problem occurs when the event rate is low compared to the sample size. Consequently, this can happen even in studies with large sample sizes.^38^

#### 2.4.2 Firth's logistic regression

Intending to reduce the bias in the ML estimator, David Firth in 1993 suggested a penalty term of $\frac{1}{2}\mathrm{trace}\left\{\frac{I{\left(\beta \right)}^{-1}\partial I\left(\beta \right)}{\partial \beta_{j}}\right\}$ in score function $U\left(\beta_{J}\right)=0$. Here, I(β) is the Fisher information matrix for β.^39^ The modified score equation will be

$$U_{M}\left(B_{j}\right)=U\left(\beta_{j}\right)+\frac{1}{2}\mathrm{trace}\left\{\frac{I{\left(\beta \right)}^{-1}\partial I\left(\beta \right)}{\partial \beta_{j}}\right\}=0\left(j=0,\ldots ,k\right).$$

The penalized log‐likelihood function related to the above‐modified score function is

$$l^{*}\left(\beta \right)=l\left(\beta \right)+\frac{1}{2}\log\left|l\left(\beta \right)\right|\cdot$$

The penalty term in the above formula is recognized as a Jeffreys invariant prior. It is worth noting that in the large dataset, the ML and Firth estimates will coincide since this formula's penalty term is asymptotically negligible.^40^ Firth's method is more attractive than post hoc bias corrections.^41^ Besides, it produces finite estimates of the OR and its CI. Although the CI can be acquired using the Wald or the profile likelihood methods, the profile likelihood CI is statistically preferable to the Wald CI with sparse data.^34^ In summary, Firth's logistic regression reduces bias in ML estimates of coefficients.^40^ In other words, this method produces finite estimates of the parameter using the average penalized ML estimate.^42^ It should be mentioned that Firth's method can be applied not only for logistic regression models but also for other generalized linear models, especially Cox regression.^39^, ^43^ Heinze and colleagues have demonstrated that in the presence of sparse data, the penalized Firth's method performs better than the standard ML method in terms of mitigating the bias when estimating the OR.^34^, ^42^, ^43^

## 3 RESULTS

### 3.1 Characteristics of study participants

This study includes 2636 women who experience menopause naturally. The mean age of women in all three groups of menopausal age was about 53 years. As shown in Table 1, about 65% of women had low or moderate socioeconomic status, these individuals were more likely to experience early menopause (*p* = 0.015). Only 5% of these women were smokers. Moreover, about 70% of women lived in rural areas, and about 22% of women were employed, with early menopause being more common in these two groups. The mean of BMI in women was approximately 26.8, with women who experienced early menopause having the lowest BMI (*p* = 0.068). Furthermore, the majority of women in this study had limited educational attainment, with those experiencing early menopause exhibiting the highest literacy level, averaging 1.84 years.

**Table 1:** The baseline characteristics of postmenopausal women of Kharameh according to age at natural menopause.

Variable	Total (*N*) = 2636	Age at natural menopause			*p* value
		Early = 549 (20.8%)	Moderate = 1512 (57.4%)	Late = 575 (21.8%)	
Socioeconomic status					
Low	848 (32.2%)	208 (37.9%)	470 (31.1%)	170 (29.6%)	0.015
Moderate	868 (32.9%)	183 (33.3%)	488 (32.3%)	197 (34.3%)	
High	624 (23.7%)	106 (19.3%)	380 (25.1%)	138 (24.0%)	
Very high	296 (11.2%)	52 (9.5%)	174 (11.5%)	70 (12.2%)	
Smoking					
Yes	141 (5.3%)	34 (6.2%)	67 (4.4%)	40 (7.0%)	0.045
No	2494 (94.6%)	515 (93.8%)	1445 (95.6%)	534 (93.0%)	
Residence					
City	807 (30.6%)	131 (23.9%)	488 (32.3%)	188 (32.7%)	0.001
Village	1829 (69.4%)	418 (76.1%)	1024 (67.7%)	387 (67.3%)	
Job					
Employee	577 (21.9%)	149 (27.1%)	321 (21.2%)	107 (18.6%)	0.002
Housewife	2059 (78.1%)	400 (72.9%)	1191 (78.8%)	468 (81.4%)	
Age	53.48 ± 8.59	53.74 ± 8.68	53.37 ± 8.64	53.53 ± 8.35	0.674
BMI	26.81 ± 4.39	26.48 ± 4.30	26.84 ± 4.43	27.09 ± 4.35	0.068
Education years	1.65 ± 2.77	1.84 ± 2.90	1.80 ± 2.88	1.08 ± 2.22	<0.001

Abbreviation: BMI, body mass index.

### 3.2 ANM and incidence of chronic diseases

**Figure 1:**
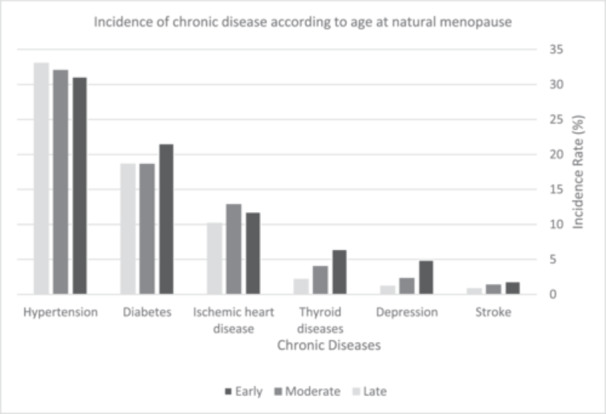
Incidence of chronic diseases according to age at natural menopause in postmenopausal women of Kharameh.

The frequency and incidence rate of each disease, along with the mean of ANM for each disease, are presented in Figure 1 and Table 2. Table 2 illustrates that the mean of ANM tends to be lower among patients with diabetes, hypertension, ischemic heart disease, stroke, thyroid disorders, and depression. It was observed that the odds of ischemic heart disease in women with early and intermediate menopause are 1.61 times (OR = 1.61, 95% CI: 1.08–2.42; *p* = 0.020) and 1.57 times (OR = 1.57, 95% CI: 1.13–2.21; *p* = 0.008) higher than in women with late menopause (Table 3).

**Table 2:** Associations between age at natural menopause and risk of chronic diseases including hypertension, diabetes, ischemic heart diseases, stroke, thyroid diseases, and depression in postmenopausal women of Kharameh.

			Total incidence rate	Total mean of ANM	ANM groups											
					Early				Moderate				Late			
					Incidence rate	Mean of ANM	95% CI		Incidence rate	Mean of ANM	95% CI		Incidence rate	Mean of ANM	95% CI	
Diseases	Status	Number					Lower	Upper			Lower	Upper			Lower	Upper
Diabetes	yes	466	19.31%	48.19	21.54%	39.79	33.18	46.41	18.67%	48.95	44.04	53.87	18.70%	56.54	48.19	61.29
	no	1947		48.75		39.94	32.58	47.30		49.25	44.32	54.18		56.58	48.75	61.37
Hypertension	yes	711	31.95%	48.38	31.08%	39.86	32.97	46.75	32.07%	48.92	43.87	53.97	33.10%	56.46	48.38	61.28
	no	1514		48.42		39.87	32.52	47.22		49.27	44.42	54.12		56.44	48.42	60.84
Ischemic heart disease	yes	305	12.10%	48.31	11.76%	39.42	32.35	46.49	12.90%	48.95	44.19	53.70	10.25%	56.63	48.31	61.47
	no	2215		48.82		40.04	32.92	47.16		49.25	44.33	54.18		56.58	48.82	61.40
Stroke	yes	36	1.38%	47.50	1.82%	40.70	35.23	46.17	1.40%	49.05	44.82	53.27	0.89%	54.60	47.50	56.35
	no	2572		48.91		39.96	32.77	47.15		49.26	44.34	54.18		56.61	48.91	61.48
Thyroid	yes	104	4.16%	47.35	6.42%	40.21	32.78	47.63	4.05%	49.62	45.02	54.22	2.23%	56.58	47.35	61.77
	no	2396		48.94		39.92	32.77	47.07		49.24	44.30	54.19		56.63	48.94	61.50
Depression	yes	67	2.70%	45.91	4.88%	38.85	30.72	46.97	2.34%	49.32	43.74	54.90	1.25%	55.57	45.91	59.93
	no	2477		49.00		40.01	32.93	47.08		49.25	44.36	54.14		56.63	49.00	61.50

Abbreviations: ANM, age at natural menopause; CI, confidence interval.

**Table 3:** Identified factors associated with ischemic heart disease using classic multiple logistic regression among postmenopausal women of Kharameh.

Variables	Number	Coefficient	OR	95 CI for exposure		*p* value
				Lower	Upper	
Menopause						
Early	544	0.479	1.614	1.080	2.422	0.020
Moderate	1449	0.453	1.573	1.132	2.215	0.008
Late	527	Reference				
Diabetes						
No	1879	Reference				
Yes	641	0.293	1.341	1.023	1.750	0.032
Hypertension						
No	1477	Reference				
Yes	1043	1.687	5.401	4.064	7.259	<0.001
Smoking						
No	2385	Reference				
Yes	135	0.502	1.652	0.983	2.685	0.049
Job						
Housewife	1961	Reference				
Employee	559	−0.607	0.545	0.367	0.788	0.002
Education years		−0.069	0.933	0.883	0.982	0.011

Abbreviations: CI, confidence interval; OR, odds ratio.

The odds of stroke in women with early menopause is 3.00 times that of women with late menopause (OR = 3.00, 95% CI: 1.08–9.32; *p* = 0.034). Furthermore, it was 81% higher in women with intermediate menopause than those with late menopause (*p* = 0.194) (Table 4).

**Table 4:** Identified factors associated with stroke using Firth's multiple logistic regression among postmenopausal women of Kharameh.

Variables	Number	Coefficient	OR	95 CI for exposure		
				Lower	Upper	*p* value
Menopause						
Early	548	1.101	3.006	1.083	9.321	0.034
Moderate	1498	0.596	1.814	0.752	5.204	0.194
Late	562	Reference				
Hypertension						
No	1505	Reference				
Yes	1103	1.410	4.095	1.955	9.512	<0.001
Residence						
City	795	Reference				
Village	1813	−0.743	0.476	0.239	0.954	0.037
Job						
Housewife	2036	Reference				
Employee	572	−0.663	0.515	0.137	1.408	0.214
Education years		−0.159	0.853	0.702	0.993	0.039

Abbreviations: CI, confidence interval; OR, odds ratio.

Diabetes and hypertension are risk factors for cardiovascular diseases, which were included in the model of ischemic heart disease and stroke to control their effect. The odds of ischemic heart disease was 5.40 times (OR = 5.40, 95% CI: 4.06–7.26; *p* < 0.001) higher in hypertensive and 1.34 times (OR = 1.34, 95% CI: 1.02–1.75); *p* = 0.032) higher in diabetic women. The occurrence of stroke was also 4.09 times higher in hypertensive women (OR = 4.09, 95% CI: 1.95–9.51; *p* < 0.001).

According to Table 5, women with early menopause were 1.46 times more likely to develop diabetes than women with late menopause (OR = 1.46, 95% CI: 1.07–2.00; *p* = 0.018). Women who experienced menopause at an intermediate age also had 10% higher odds of developing diabetes; Although this relationship was not significant (*p* = 0.481). As shown in Table 6, Early and intermediate menopause also increased the odds of developing hypertension, but this relationship was not significant (*p* = 0.495 and *p* = 0.806, respectively).

**Table 5:** Identified factors associated with diabetes using classic multiple logistic regression among postmenopausal women of Kharameh.

Variables	Number	Coefficient	OR	95 CI for exposure		
				Lower	Upper	*p* value
Menopause						
Early	534	0.378	1.460	1.067	2.00	0.018
Moderate	1387	0.097	1.101	0.845	1.446	0.481
Late	492	Reference				
Residence						
City	711	Reference				
Village	1702	−0.453	0.636	0.504	0.802	<0.001
Job						
Housewife	1862	Reference				
Employee	551	−0.660	0.517	0.383	0.688	<0.001
BMI		0.032	1.033	1.009	1.057	0.007
Education years		−0.126	0.882	0.839	0.924	<0.001

Abbreviations: BMI, body mass index; CI, confidence interval; OR, odds ratio.

**Table 6:** Identified factors associated with hypertension using classic multiple logistic regression among postmenopausal women of Kharameh.

Variables	Number	Coefficient	OR	95 CI for exposure		
				Lower	Upper	*p* value
Menopause						
Early	518	0.099	1.104	0.832	1.466	0.495
Moderate	1275	0.030	1.030	0.812	1.312	0.806
Late	432	Reference				
Residence						
City	652	Reference				
Village	1573	−0.555	0.574	0.464	0.710	<0.001
Job						
Housewife	1711	Reference				
Employee	514	−0.620	0.538	0.419	0.686	<0.001
BMI		0.052	1.054	1.031	1.076	<0.001
Education years		−0.134	0.874	0.838	0.910	<0.001

Abbreviations: BMI, body mass index; CI, confidence interval; OR, odds ratio.

The odds of depression in women with early menopause was 4.79 times that of women with late menopause (OR = 4.79, 95% CI: 2.20–11.79; *p* = <0.001). In addition, women with intermediate menopause were 83% more prone to depression (*p* = 0.114) (Table 7).

**Table 7:** Identified factors associated with depression using Firth's multiple logistic regression among postmenopausal women of Kharameh.

Variables	Number	Coefficient	OR	95 CI for exposure		
				Lower	Upper	*p* value
Menopause						
Early	533	1.567	4.793	2.197	11.794	<0.001
Moderate	1452	0.607	1.835	0.872	4.408	0.114
Late	559	Reference				
Residence						
City	770	Reference				
Village	1774	−1.014	0.363	0.218	0.597	<0.001
Job						
Housewife	1980	Reference				
Employee	564	−0.818	0.441	0.174	0.944	0.034
BMI		0.050	1.051	0.994	1.111	0.082

Abbreviations: BMI, body mass index; CI, confidence interval; OR, odds ratio.

**Figure 2:**
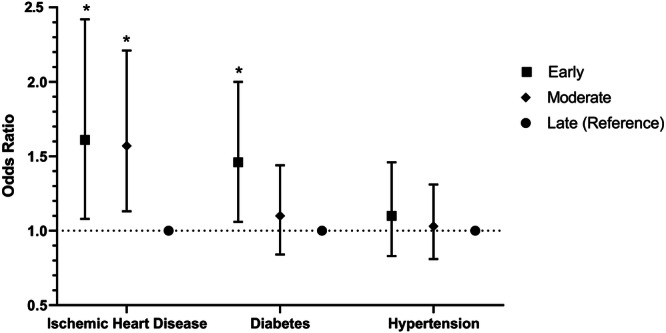
Associations between age at natural menopause and the risk of ischemic heart diseases, diabetes, and hypertension in postmenopausal women of Kharameh.

**Figure 3:**
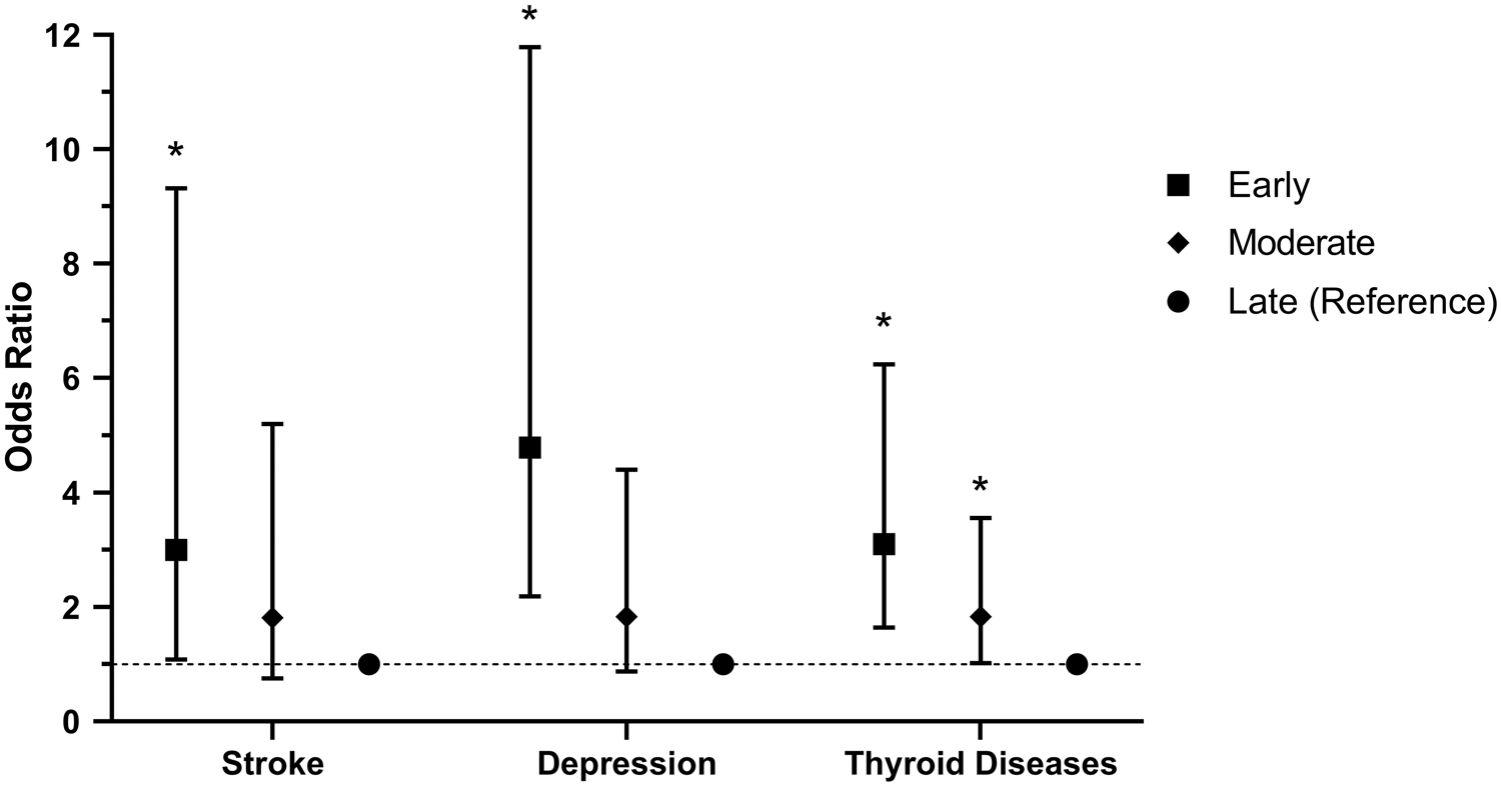
Associations between age at natural menopause and the risk of stroke, depression, and thyroid diseases in postmenopausal women of Kharameh.

Compared to women with late menopause, women with early and intermediate menopause were 3.10 times (OR = 3.10, 95% CI: 1.64–6.24; *p* < 0.001) and 1.83 times (OR = 1.83, 95% CI: 1.02–3.57; *p* = 0.042) more likely to develop thyroid diseases (Table 8). Figures 2 and 3 shows the odds ratio and confidence intervals related to the association between ANM and chronic diseases.

**Table 8:** Identified factors associated with thyroid diseases using Firth's multiple logistic regression among postmenopausal women of Kharameh.

Variables	Number	Coefficient	OR	95 CI for exposure		
				Lower	Upper	*p* value
Menopause						
Early	530	1.132	3.103	1.644	6.242	<0.001
Moderate	1431	0.607	1.835	1.021	3.569	0.042
Late	539	Reference				
Smoking						
No	2363	Reference				
Yes	137	0.692	1.998	0.885	3.997	0.091
Residence						
City	748	Reference				
Village	1752	−0.590	0.554	0.368	0.839	0.006
Age		0.028	1.028	1.005	1.052	0.016

Abbreviations: CI, confidence interval; OR, odds ratio.

Rural dwellers were less likely to develop depression (OR = 0.36, 95% CI: 0.22–0.60; *p* < 0.001), diabetes (OR = 0.64, 95% CI: 0.50–0.80; *p* < 0.001), hypertension (OR = 0.57, 95% CI: 0.46–0.71; *p* < 0.001), thyroid (OR = 0.55, 95% CI: 0.37–0.840; *p* = 0.006), and stroke (OR = 0.48, 95% CI: 0.24–0.95; *p* = 0.037). In the ischemic heart disease model, this relationship was not significant due to the correlation and collinearity of residence with ANM (*p* < 0.001).

Being employed was a protective factor for diabetes (OR = 0.52, 95% CI: 0.38–0.69; *p* < 0.001), hypertension (OR = 0.54, 95% CI: 0.42–0.69; *p* < 0.001), depression (OR = 0.44, 95% CI: 0.17–0.94; *p* = 0.034), and ischemic heart diseases (OR = 0.54, 95% CI: 0.37–0.79; *p* = 0.002).

BMI had a positive relationship with diabetes (OR = 1.03, 95% CI: 1.009–1.057; *p* = 0.007) and hypertension (OR = 1.05, 95% CI: 1.03–1.08; *p* < 0.001).

With each year of increasing education, the odds of diabetes, hypertension, ischemic heart disease, and stroke decrease by 12% (OR = 0.88, 95% CI: 0.84–0.92; *p* < 0.001), 13% (OR = 0.87, 95% CI: 0.84–0.91; *p* < 0.001), 7% (OR = 0.93, 95% CI: 0.88–0.98; *p* = 0.011) and 15% (OR = 0.85, 95% CI: 0.70–0.99; *p* = 0.039), respectively.

Although smokers were 1.65 times (OR = 1.65, 95% CI: 0.98–2.68; *p* = 0.049) and 2 times (OR = 2.00, 95% CI: 0.88–4.00; *p* = 0.091) more likely to suffer from ischemic heart disease and thyroid diseases, respectively; this relationship was not significant.

## 4 DISCUSSION

The age of menopause determines the health status and risk of future diseases in women.^44^ This survey aimed to investigate the relationship between ANM and the incidence of some common chronic diseases in the postmenopausal women of Kharameh, located in the south of Iran. Given the findings, younger ANM increases the odds of developing ischemic heart diseases, stroke, diabetes, depression, and thyroid diseases (Figure 1).

Our study demonstrated that the odds of stroke and ischemic heart disease is higher in women with early and intermediate menopause. However, in some studies, there was no relationship between the age of menopause and cardiovascular diseases.^16^ Shen et al.^45^ observed that for every year delay in menopause, the prevalence of coronary heart disease and stroke decreases by 3% (OR: 0.97; 95% CI: 0.95–0.98) and 5% (OR: 0.95; 95% CI: 0.95–0.98), respectively. Increased risk of ischemic heart diseases in women with early menopause has been reported in other investigations too.^46^, ^47^

We found that occurrence of menopause before 45 years old is a risk factor for stroke. Similarly, in the Framingham Heart Study, ANM before age 42 was associated with increased ischemic stroke risk.^48^ However, according to another study conducted on 5731 naturally postmenopausal women more than 65 years old, there was no significant association between stroke and menopause age.^49^ Indeed, one of the reasons for the increased risk of cardiovascular diseases after menopause is the higher androgen/estrogen ratio in postmenopausal women compared to women who have not yet reached menopause.^50^ Estrogens have a protective role on the cardiovascular system through direct action on the vascular wall (increasing vasodilation, inhibiting the response to vascular damage), improving lipid profile and insulin sensitivity, and increasing peripheral fat deposition.^51^ After menopause, the level of sex hormone‐binding globulin (SHBG) decreases, leading to relatively higher levels of testosterone or a higher free androgen index.^52^ High androgen concentration and low SHBG concentration are associated with cardiovascular disease risk factors after menopause.^53^

In this study, the odds of ischemic heart disease was higher in women who suffered from diabetes and hypertension, as well as those who had low education and were housewives. In past studies, diabetes and hypertension have been introduced as risk factors for ischemic heart diseases.^54^ In addition, it has been observed that being housewives^54^ and less educated^55^, ^56^ are risk factors for ischemic heart diseases. Given our results, women with hypertension, low education, housewives, and city dwellers were more prone to stroke. In line with our findings, former studies have indicated that stroke is directly associated with hypertension,^57^, ^58^ living in the city,^59^, ^60^ and inversely with education^61^, ^62^ and employment.^63^

According to our detections, women who experienced early menopause were more likely to develop diabetes than women who experienced late menopause. In line with our results, another study conducted on 4968 postmenopausal women from the National Health and Nutrition Examination Survey 2011–2018, found an association between age at menopause of <40 years and increased risk of type 2 diabetes mellitus.^64^ However, Lee et al.^31^ in their retrospective study and Que et al.^65^ in their cross‐sectional study found no relationship between age at menopause and diabetes mellitus risk. One of the mechanisms that can increase the risk of diabetes in postmenopausal women is shorter exposure to estrogen, which may have a protective role in the function of pancreatic β cells.^66^ Low postmenopausal SHBG concentrations are associated with central obesity and insulin resistance.^67^

Furthermore, housewives, city dwellers, less educated, and women with higher BMI were at greater risk of developing diabetes. These results have also been observed in a study conducted on the Kharamah population.^68^ In other studies, the relationship between diabetes and higher BMI,^69^ living in urban areas,^69^, ^70^ being a housewife,^71^ and low education^69^, ^71^ have been observed.

The prevalence of hypertension, which is one of the main risk factors for cardiovascular diseases, is lower in non‐menopausal women than in men, while it is higher in postmenopausal women than in men.^72^ As mentioned, menopause is associated with a change in the estrogen/androgen ratio. The difference in blood pressure between men and women is due to estrogen's protective role or testosterone's threatening role.^73^ Several mechanisms can contribute to postmenopausal hypertension, such as endothelial dysfunction, inappropriate activation of renin‐angiotensin and sympathetic systems, oxidative stress, inflammatory mediators, dyslipidemia, and weight gain.^74^, ^75^, ^76^ In Song et al.'s^77^ study, women who reached menopause at the age of 45 or less and 46–52 years had 1.27 times and 1.14 times higher chance of developing hypertension compared to those who reached menopause at the age of at least 53 years. However, in line with our study, some investigations didn't observe any significant association between ANM and hypertension.^16^, ^18^

This survey revealed that housewives, less educated, city dwellers, and with higher BMI had higher odds of developing hypertension. In other studies, hypertension has been associated with higher BMI,^78^, ^79^ lower education,^80^ living in urban areas,^70^ and unemployment conditions.^80^

Given the results, those who experienced early menopause were more likely to develop depression in later years than those who experienced late menopause. In past studies, early menopause has been associated with an increased risk of depression, which is attributed to less exposure to endogenous estrogens that have neuroprotective and antidepressant properties.^24^, ^81^, ^82^, ^83^ In addition, women who were housewives, lived in the city and had higher BMI were more likely to suffer from depression. In line with our results, in other studies, there was a direct relationship between depression and living in the city^84^ and an inverse relationship with employment.^84^

The thyroid gland undergoes important functional changes during aging and simultaneously the prevalence of thyroid disorders increases with age.^85^ The results of this study also showed that the incidence of thyroid diseases increases with age. In addition, it was observed that women who experience early and moderate menopause are more likely to develop thyroid diseases than those who experience late menopause. Consistent with our results, Zamanian et al.,^27^ observed that the age of menopause is significantly lower in women with thyroid disease, although no significant relationship was observed in multiple regression. In another study conducted by Amiri et al.,^28^ a significant association was observed between thyroid diseases and the early age of menopause. However, in another research, there was no relationship between thyroid disease and natural menopause age.^86^ Overall, studies on the relationship between menopause and thyroid function are few, and the effect of menopause on thyroid diseases regardless of age has not been determined.^87^

We discussed the effect of some hormonal and biological changes during menopause on chronic diseases but the role of behavioral factors should not be neglected. For example, some women may experience changes in lifestyle behaviors during menopause, such as decreased physical activity, poor diet, and increased stress, which can further contribute to the development of chronic diseases.^88^

The effect of menopausal age on chronic disease development has significant practical, clinical, and public health implications. Women who experience premature menopause may have an increased risk of developing chronic conditions and multimorbidity.^1^ Early identification of women with early menopause can help prevent chronic diseases by providing timely and appropriate interventions including lifestyle modifications such as healthy diet, exercise, screening and risk assessment, and other aspects of health care. Understanding the underlying mechanisms and risk factors associated with early menopause can help healthcare providers develop targeted prevention and management strategies to reduce the risk of chronic diseases in women experiencing early menopause.

The strength of this historical cohort is the large population of participants who entered the study through the census, it is worth mentioning that 98.4% of the eligible people of Kharameh agreed to participate in the Kharameh cohort study. In this study, to make a more accurate estimate, Firth's logistic regression approach was used for rare diseases. Howbeit, it is a retrospective design and cannot confirm the causal relationship between age at menopause and the study outcomes. furthermore, only the participants who were willing to fill out the questionnaires regarding menopause were included, which might have led to selection bias. Information relied on self‐reports and may have been subject to recall bias and reporting bias, resulting in misclassification of the menopausal status. There may be residual confounders that could not be considered due to lack of data. Since this study was conducted on women aged 40–70 years, we were not able to consider postmenopausal women under 40 and over 70 years. Although it is almost proven that hormonal changes can cause some diseases, we can cautiously generalize our results to general Iranian postmenopausal women because Iran is a vast country with cultural, genetic, and lifestyle diversity that makes women from different regions susceptible to some diseases.

Based on the diseases investigated in this society, it is beneficial for women to have late ANM. But some people are more susceptible to some diseases due to different genetic backgrounds so we cannot make a definite prescription for all women. Further exhaustive and multicenter studies encompassing a wider spectrum of diseases are necessary to come to a definitive conclusion. In addition, further research is needed to determine the most effective lifestyle interventions for preventing chronic diseases in women with early menopause such as exercise programs, stress‐reducing techniques, and hormone replacement therapy.

## 5 CONCLUSION

In this study, early and intermediate menopause were associated with ischemic heart disease and thyroid diseases, while early menopause was also a risk factor for diabetes, depression, and stroke. These findings highlight the importance of monitoring women's health during and after menopause, and may help inform preventative measures and treatment strategies for these chronic diseases.

## AUTHOR CONTRIBUTIONS

**Zahra Pasokh**: conceptualization; formal analysis; investigation; software; visualization; writing—original draft. **Mozhgan Seif**: conceptualization; methodology; project administration; software; writing—review & editing. **Haleh Ghaem**: conceptualization; writing—review & editing. **Abbas Rezaianzadeh**: data curation; resources; supervision; validation. **Masoumeh Ghoddusi Johari**: investigation.

## CONFLICT OF INTEREST STATEMENT

The authors declare no conflicts of interest.

## ETHICS STATEMENT

This study was approved by the Ethics Committee of Shiraz University of Medical Sciences (IR.SUMS.SCHEANUT.REC.1400.104).

## TRANSPARENCY STATEMENT

The lead author Mozhgan Seif affirms that this manuscript is an honest, accurate, and transparent account of the study being reported; that no important aspects of the study have been omitted; and that any discrepancies from the study as planned (and, if relevant, registered) have been explained.

## Data Availability

The data that support the findings of this study are available from the corresponding author upon reasonable request.
